# A Rare Case of Autopsy Proven Achromobacter xylosoxidans Endocarditis Involving Tricuspid Valve: A Case Report

**DOI:** 10.7759/cureus.38118

**Published:** 2023-04-25

**Authors:** Chaitra Janga, Mackenzie Kramer, Ifrah Naeem, Lucy Checchio, Zahra Qamar

**Affiliations:** 1 Internal Medicine, Jefferson Health-Abington, Abington, USA; 2 Infectious Disease, Jefferson Health-Abington, Abington, USA

**Keywords:** surgery, iv antibiotics, tricuspid valve endocarditis, achromobacter xylosoxidans, infective endocarditis

## Abstract

Infective endocarditis refers to infection of one or more valves of the heart, with *Achromobacter xylosoxidans* (*A. xylosoxidans*) being a rare cause. So far, 24 cases of *A. xylosoxidans* endocarditis were reported, with only one case describing tricuspid valvular involvement. Despite the rarity of *A. xylosoxidans* endocarditis, it is important for clinicians to be aware of atypical presentation and the high mortality associated with it. We present an autopsy-proven case of tricuspid valve endocarditis in the setting of *A. xylosoxidans* bacteremia in a 43-year-old female.

## Introduction

This article was previously presented as a meeting abstract at the 2022 CHEST Annual Scientific Meeting in October 2022.

*Achromobacter xylosoxidans* (*A. xylosoxidans*) is a Gram-negative rod, usually present in aqueous environments, and has high mortality rates. This organism is more commonly associated with nosocomial infections and seldom causes endocarditis. The rarity of this organism and its high resistance pattern limit successful treatment modalities. While there is no current consensus on management, recommendations include a combination of antibiotic therapy and valve replacement surgery [1].

## Case presentation

A 43-year-old African American female with a past medical history significant for lymphedema and recent pulmonary embolism (low risk) on Apixaban presented in septic shock. Blood cultures were obtained on day one of admission to the medical intensive care unit; positive for *A. xylosoxidans*, which were susceptible to Meropenem, Piperacillin-Tazobactam, and Trimethoprim-Sulfamethoxazole. Imaging did not show any evidence of infection, and the echocardiogram was negative for vegetation. The source potentially responsible for septic shock was her lower extremity wounds. During her hospitalization of 10 days, four subsequent sets of blood cultures remained negative. She received a total of two weeks of Piperacillin-Tazobactam and Vancomycin and was subsequently discharged.

Two months later, the patient was re-admitted for chest pain and altered mental status. On initial presentation, vitals were temperature of 98.4°F, tachycardic at 132 bpm, blood pressure 70/48 mmHg, respiratory rate 25/min, and saturating at 100% on room air. Hypotension persisted even after aggressive fluid resuscitation and she was eventually started on a pressor medication. Significant laboratory data are shown in Table 1. On physical examination, the patient was confused, tachycardic with a regular rhythm, and lung auscultation revealed normal breath sounds. Skin examination showed lower extremity discoloration with healed wounds and pitting edema. Initially, the patient was started on Piperacillin-Tazobactam. Again, blood cultures grew *A. xylosoxidans* susceptible to Ceftazidime, Meropenem, Piperacillin-Tazobactam, and Trimethoprim-Sulfamethoxazole. Due to suspicion of meningitis, antibiotics were broadened to Meropenem for central nervous system (CNS) penetration. CT scan of the head was normal without any acute intracranial pathology. CT scan of her lower extremity showed diffuse soft tissue inflammation, suggestive of cellulitis. CT chest/abdomen/pelvis was performed to look for other sources of infection, negative except for septic pulmonary emboli. Echocardiography revealed a 13 mm x 9.6 mm echodensity on the anterior leaflet of the tricuspid valve (Video 1), with normal left and right ventricular systolic function without segmental wall motion abnormalities. For suspected tricuspid valve infective endocarditis secondary to *A. xylosoxidans*, Cardiovascular-Thoracic surgery was consulted. However, the patient was hemodynamically unstable for surgical intervention due to requiring intubation for airway protection, and increasing doses of vasopressor medications for blood pressure support. Despite the above aggressive measures, she remained acidotic, was started on continuous renal replacement therapy, and eventually succumbed to her disease. The source responsible for her bacteremia was still unknown as there were no procedures performed including central line placement. Autopsy showed a 1.0 cm vegetation on the tricuspid valve, bilateral acute pneumonia (right and left lower lobes), multiple septic emboli, and diffuse alveolar damage. Histopathology of the tricuspid valve showed acute inflammation, organized thrombus, and embedded bacterial colonies. 

**Table 1:** Laboratory data. WBC, white blood cell; AST, aspartate aminotransferase; ALT, alanine transaminase; ESR, erythrocyte sedimentation rate; CRP, C-reactive protein; INR, international normalized ratio; BNP, brain natriuretic peptide

Lab value	Result	Reference
Hemoglobin	11.1	14-17 g/dL
WBC	20.6	4-8 b/L
Platelet	174	140-400 b/L
Sodium	129	135-146 mmol/L
Bicarb	10	21-30 mmol/L
Anion gap	23	6-14 mmol/L
Creatinine	4.62	0.70-1.40 mg/dL
AST	41	7-42 IU/L
ALT	37	<45 IU/L
Lactate	12.2	0.5-1.6 mmol/L
ESR	76	0-20 mm/h
CRP	35	<=0.80 mg/dL
INR	1.4	0-84-1.16
Troponin Ths	19	< 19 g/L
pro BNP	15077	< 450 pg/mL

**Video 1:** Video clip showing echodensity on anterior leaflet of tricuspid valve.

## Discussion

*Achromobacter xylosoxidans *is an aerobic motile, Gram-negative rod that is usually associated with central line infections and seldom causes endocarditis. Literature has shown about 24 cases of infective endocarditis caused by *A. xylosoxidans*, with rare involvement of the tricuspid valve (Table 2). Among the cases reported, mortality is high at about 48%, with risk factors being prosthetic devices and impaired immunity [1-24]. 

**Table 2:** Reported cases of Achromobacter xylosoxidans endocarditis. AF, atrial fibrillation; CKD, chronic kidney disease; COPD, chronic obstructive pulmonary disease; HF, heart failure; H, hypertension; DM, diabetes mellitus; CABG, coronary artery bypass grafting; CS, cardiac surgery; CVC, central venous catheter; IHD, ischemic heart disease; IS, ischemic stroke; MS, mitral stenosis; NA, not available; NR, not reported; PE, pulmonary embolism; PM, pacemaker; PrV, prosthetic valve; TIA, transient ischemic attack; TMP-SMX, trimethoprim-sulfamethoxazole

S No	Author and year of publication	Age/Sex	Suspected risk factor or predisposing factor	Comorbidities	Affected valve	Valve prosthesis	Antibiotics used and duration of treatment	Surgical Intervention	Overall outcome
1.	Joseph et al. (2022) [1]	81 Y/ F	Mitral rheumatic aortic stenosis	AF, acute rheumatic fever	Mitral	No	Piperacillin/tazobactam + TMP-SMX; 6 weeks	No	Died
2.	GianFranco et al. (2022) [2]	57 Y/ M	CVC	high-grade B-cell Non-Hodgkin lymphoma	Aortic	No	Cefiderocol + Fosfomycin+ TMP-SMX; 3 weeks	None	Survived
3.	De Castro et al. (2021) [3]	19 Y/ M	CS, aortic bicuspid	None	Aortic	No	Carbapenem; 4 weeks	Yes	Survived
4.	Tea et al. (2019) [4]	67 Y/ M	Rheumatic mitral stenosis	Iatrogenic asplenia	Mitral valve	No	Piperacillin-sulbactam + Imipinem, 12 weeks	Yes, MVR	Survived
5.	Xia et al. (2018) [5]	66 Y/ F	Venous catheter	H, DM, CKD	Mitral and aortic	No	Merpenem, Vancomycin, Levofloxacin, TMP-SMX; NR	No	Died
6,	Rodrigues et al. (2017) [6]	86 Y/ F	NR	Pulmonary fibrosis, IHD, CKD	Aortic	No	Piperacillin-tazobactam + TMP-SMX; 6 weeks	No	Survived
7.	Kumar et al. (2017) [7]	54 Y/ M	None	CKD, H	Mitral and aortic	No	Vancomycin + piperacillin-tazobactam + gentamicin; 2 weeks	Yes	NR
8.	Bhattarai et al. (2016) [8]	37 Y/ NR	PrV	NR	Mitral	Yes	Meropenem; NR	Yes	Survived
9.	Levoy et al. (2015) [9]	6 months/ M	Venous catheter	Arterial calcification	Mitral	No	Piperacillin-tazobactam + TMP-SMX + colistin + meropenem + levofloxacin; 6 weeks	No	Survived
10.	Rafeal et al. (2014) [10]	50 Y/ F	CS	Ventricular septum surgery	Pulmonary and ventricle repair	No	Piperacillin/tazobactam+ TMP-SMX; 8 weeks	Yes	Survived
11.	Sawant et al. (2013) [11]	62 Y/ F	PrV + pacemaker	AF, HF, COPD, CABG	Mitral Aortic Pacemaker	yes/no/-	Piperacillin-tazobactam + TMP-SMX + amikacin + meropenem + rifampicin; 6 weeks	Yes	Survived
12.	Tokuyasu et al. (2012) [12]	86 Y/ F	PrV	NR	Aortic	Yes	Carbapenem; 4 weeks	No	Died
13.	Derber et al. (2011) [13]	54 Y/ F	PrV + Fallot’s tetralogy	Fallot’s tetralogy	Pulmonary	Yes	Piperacillin-tazobactam + imipenem-cilastatin, levofloxacin; 8 weeks	Yes	Survived
14.	Storey et al, (2010) [14]	79 Y/ F	None	AF, TIA, H	Mitral and aortic	No	Meropenem; 6 weeks	No	Died
15.	Ahmed et al. (2009) [15]	69 Y/ M	PrV	DM, CABG, H	Mitral and aortic	Yes for aortic only	Ertapenem + tigecycline + TMP/SMX; NR	Yes	Died
16.	Malek-Marin et al. (2009) [16]	50 Y/ M	Catheter	CKD	NR	NR	NR	Yes	Died
17.	Van Hal et al. (2008) [17]	37 Y/ M	PrV IHD	NR	Aortic	Yes	Carbapenem; 6 weeks	Yes	Survived
18.	Yang et al. (2007) [18]	35 Y/ M	IHD, TIA, pacemaker	Hepatitis C	Tricuspid	No	Piperacillin-tazobactam + amikacin + ceftazidime; NR	Yes	NR
19.	Nanuashvili li et al. (2007) [19]	46 Y/ M	NR	Diabetes, IS, emphysema	Mitral Aortic	NR	Ampicillin + tazobactam + cotrimoxazole; 4 weeks	Yes	Survived
20.	Ahn et al. (2004) [20]	35 Y/ M	CS pacemaker	CS	Pacemaker and ventricular repair	NR	Ceftazidime + piperacillin; 3 weeks	Yes	Survived
21.	Martino et al. (1990) [21]	33 Y/ M	Venous catheter	Bone marrow transplantation	NR	NR	Aztreonam + amikacin; 6 weeks	No	Died
22.	Davis et al. (1982) [22]	30 Y/ M	MS	MS, HF	Aortic	NR	NR	No	Died
23.	Olson et al. (1982) [23]	35 Y/ M	Aortic surgery valve	NR	Aortic	Yes	Carbenicillin + TMP-SMX + rifampicin + moxalactam + azlocillin; 15 weeks	No	Died
24.	Lofgren et al. (1981) [24]	77 Y/ F	PrV	Rheumatic heart disease PrV	Mitral and aortic	Yes for PrV only	Tobramycin + carbapenicillin + TMP-SMX + moxalactam; 3 weeks	No	Died
25.	Our case (2023)	43 Y/ F	None	PE, lymphedema	Tricuspid	No	Piperacillin-Tazobactam, Meropenem; 3 weeks	No	Died

Common risk factors include the presence of a central venous catheter (CVC) or any indwelling catheter, tunneled hemodialysis catheters, immunocompromised state (cystic fibrosis, malignancy), pre-existing valvular disease, or the presence of a prosthetic device. The mode of transmission can be from contaminated water, contaminated IV contrast, disinfectants, ultrasound gels, poor hygiene among healthcare workers, and poor adherence to infection control protocols [25]. Achromobacter’s nature to form biofilms on prosthetic material makes it exceedingly difficult to eradicate completely. Although the source of infection is unclear in our patient; recent hospitalization and contaminated medical equipment could be possibilities. 

Although work-up is similar to any typical infective endocarditis including initial evaluation with modified Duke criteria along with blood cultures, electrocardiogram (EKG), transthoracic echocardiography (TTE), transesophageal echocardiography (TEE), the atypical presentations of Achromobacter make it difficult to diagnose despite repetitive imaging and evaluations. A review from 2012 reported the challenges in the diagnosis of Achromobacter endocarditis, which was identified on the 131st day after performing four separate TTEs and TEEs [12]. This highlights the atypical nature and the need for repetitive diagnostic evaluations if suspicion for infective endocarditis is high, as in our patient. Cardiac computed tomography angiography (CTA) and positron emission tomography with computed tomography (PET/CT) are recommended if echocardiographic evaluation is inconclusive [26]. Due to PET-CT being a functional and non-invasive modality, it is helpful in detecting infection prior to valvular structural damage, and in identifying embolic and extra-cardiac sources [27]. FDG PET-CT is usually preferred to radiolabeled white blood cell single-photon emission computed tomography (WBC SPECT), due to later's low sensitivity, time consumption, and increased expertise requirements [28]. However, a negative PET-CT cannot completely rule out the infection, as reported in a review by Tokuyasu et al. [12]. Additional imaging studies include CXR, CT chest, and CT/MRI head to rule out septic emboli. 

Achromobacter is usually susceptible to antipseudomonal penicillins and Carbapenems. Studies have shown increasing resistance to bactrim and aminoglycosides [29]. Due to extensive drug resistance patterns, various regimens have been used; however, evidence on optimal regimens is still lacking [30]. In 2015, Abbott and Peleg recommended various monotherapies in comparison to combination therapies. This study also recommended the use of aminoglycosides like Gentamicin along with bactrim for a synergistic effect despite the prevalence of resistance [31]. Also, a review in 2018 described the rapid development of carbapenemase resistance due to a prolonged course of Meropenem, which led to poor outcomes [5]. A recent report in 2022 has successfully treated Achromobacter endocarditis using a Cefiderocol combination regimen [2]. In this study, the initial therapy used was a combination of Meropenem, Fosfomycin, and Trimethoprim-Sulfamethoxazole; however, due to persistent symptoms Meropenem was replaced with Cefiderocol. A significant increase in bactericidal activity was observed after this addition, which is due to Cefiderocol’s activity against biofilm-forming pathogens. 

Although there is no specific consensus on the duration of treatment, most cases reported a minimum duration of 6 weeks of treatment with close follow-ups. Despite this, there are reports of treatment failures leading to recurrent admissions and death [1]. Literature has also shown that treatment employing both antibiotics and surgical intervention demonstrated a decrease in mortality when compared to antibiotics alone, reinforcing the importance of early surgical consultation for interventions [1, 5]. Catheter removal is recommended if CVC biofilms are the suspected source.

## Conclusions

In summary, we presented a diagnostically challenging patient presenting with *A. xylosoxidans* endocarditis diagnosed after repetitive imaging and proven by autopsy. This case highlights the atypical presentation of patients with *A. xylosoxidans* bacteremia, especially endocarditis, and stresses the importance of a high suspicion in such cases. Knowledge about successful treatment of *A. xylosoxidans* is scarce, and more research is warranted for better management in the future.
